# Supplementary material for: Transplantation of GABAergic Interneuron Progenitors Restores Cortical Circuit Function in an Alzheimer's Disease Mouse Model
Source: Adv Sci (Weinh). 2025 Nov 27;13(9):e11472. doi: 10.1002/advs.202511472 (PMC12904069; doi:10.1002/advs.202511472)
Supplement: Supplementary file 1 — Supporting Information [file ADVS-13-e11472-s001.pdf]

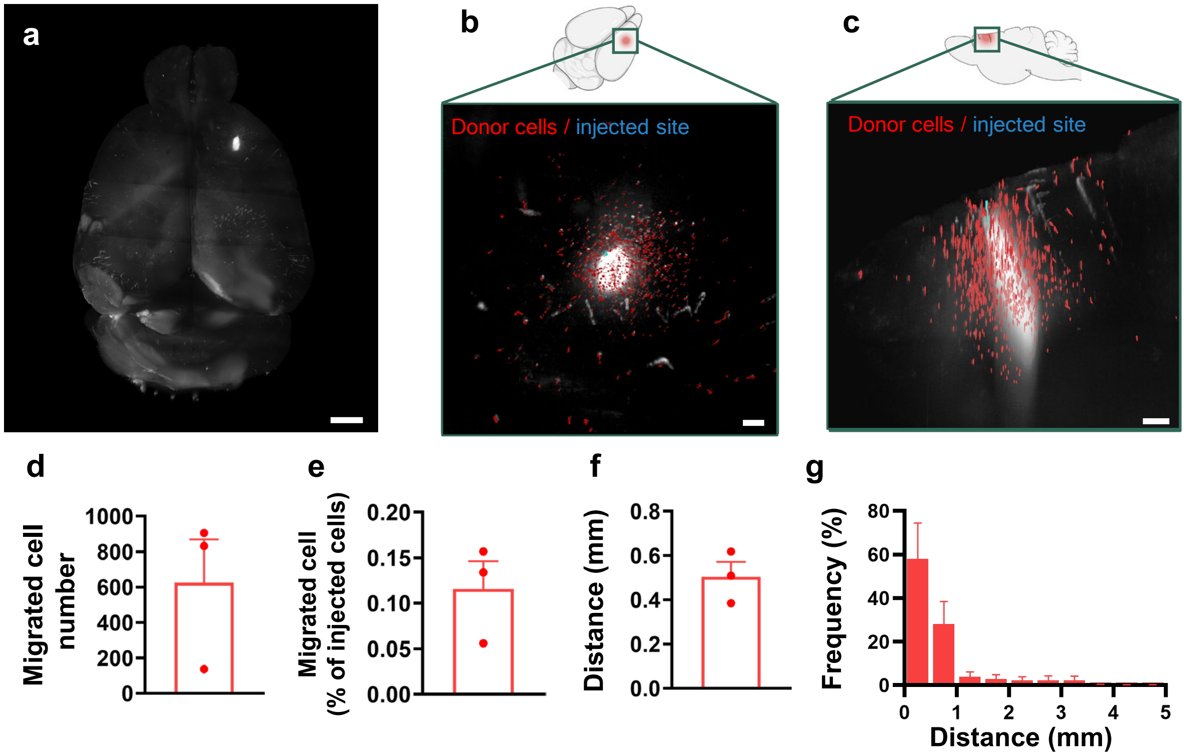

Supplementary Fig. 1 | MGE cells transplanted into wild-type cortex survive for 60 days and migrate. **a** Three-dimensional (3D) reconstruction of the entire host brain. **b, c** Higher magnification 3D reconstructions from the dorsal (**b**) and sagittal (**c**) views showing donor cells (red) and the injection site (blue). **d** Number of migrated cells detected by whole-brain imaging. **e** Percentage of donor cells that migrated, calculated by dividing the number of migrated cells by the total number of transplanted cells. **f** Mean distance traveled by donor cells, measured from the injection site. **g** Distribution of distances traveled by donor cells. Data are presented as mean ± SD. Scale bars: 1 mm (**a**), 0.2 mm (**b**), 0.3 mm (**c**). $n = 3$ mice.

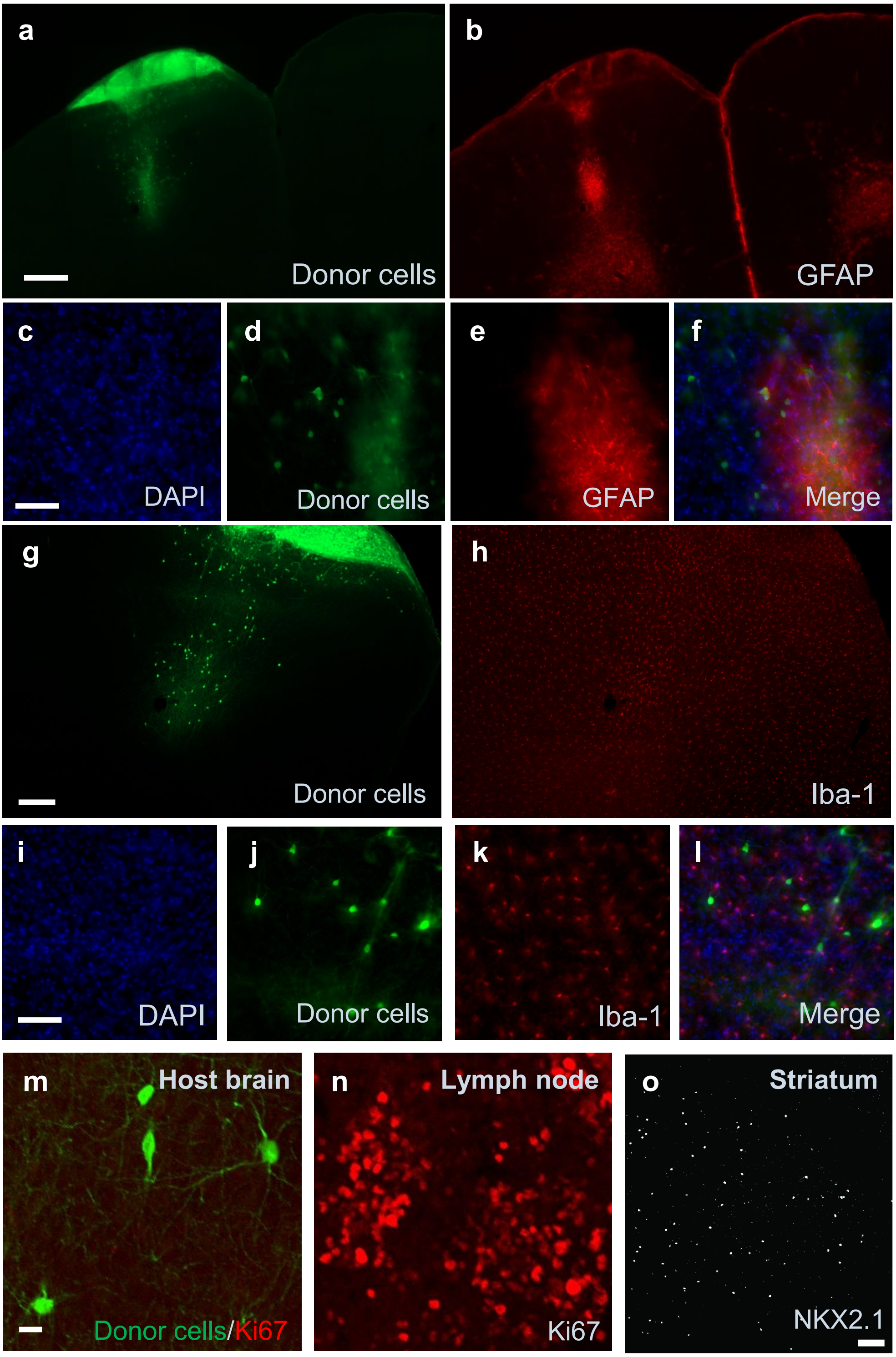

Supplementary Fig. 2 | Immunostaining revealed GFAP-positive astrocyte accumulation, but not microglial accumulation, at the injection site. a–f GFAP immunostaining in the host anterior neocortex 2 months post-transplantation. **g–l** Iba-1 immunostaining in the host anterior neocortex 2 months post-transplantation. **m** Ki67 immunostaining in the host anterior neocortex 2 months post-transplantation. **n–o** Staining quality controls: lymph node for Ki67 (**n**) and striatum for NKX2.1 (**o**). Scale bars: 400 µm (**a**), 50 µm (**c, i**), 20 µm (**m**), and 100 µm (**o**).

a

	#
Mice examined	13
Images (sections) examined	69
Donor cells with long-range processes (>400 µm)	6
Donor processes beneath corpus callosum	11
Donor cell bodies beneath corpus callosum	2
Donor processes in contralateral hemispheres	1
Donor cells bodies in contralateral hemispheres	0

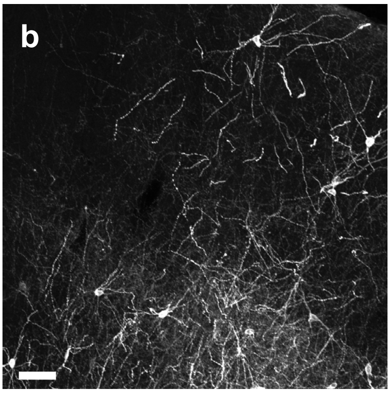

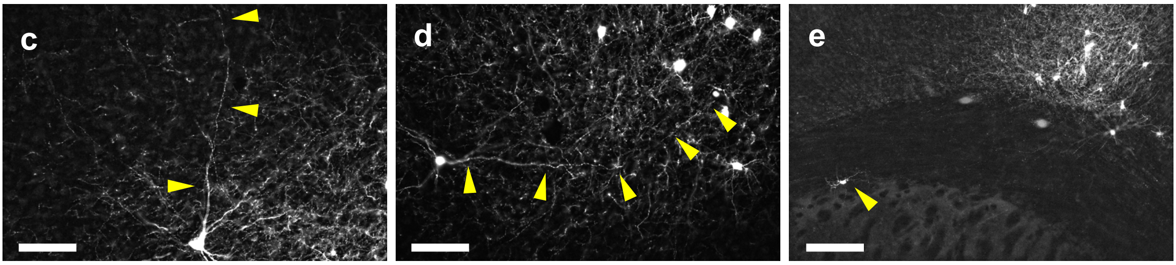

Supplementary Fig. 3 | The neurites of MGE donor cells were broadly distributed. a Sample and counting results**. b–e** GFP immunostaining in the host anterior neocortex 2 months post-transplantation. Scale bars: 120 µm (**a**), 100 µm (**c, d**), and 200 µm (**e**).

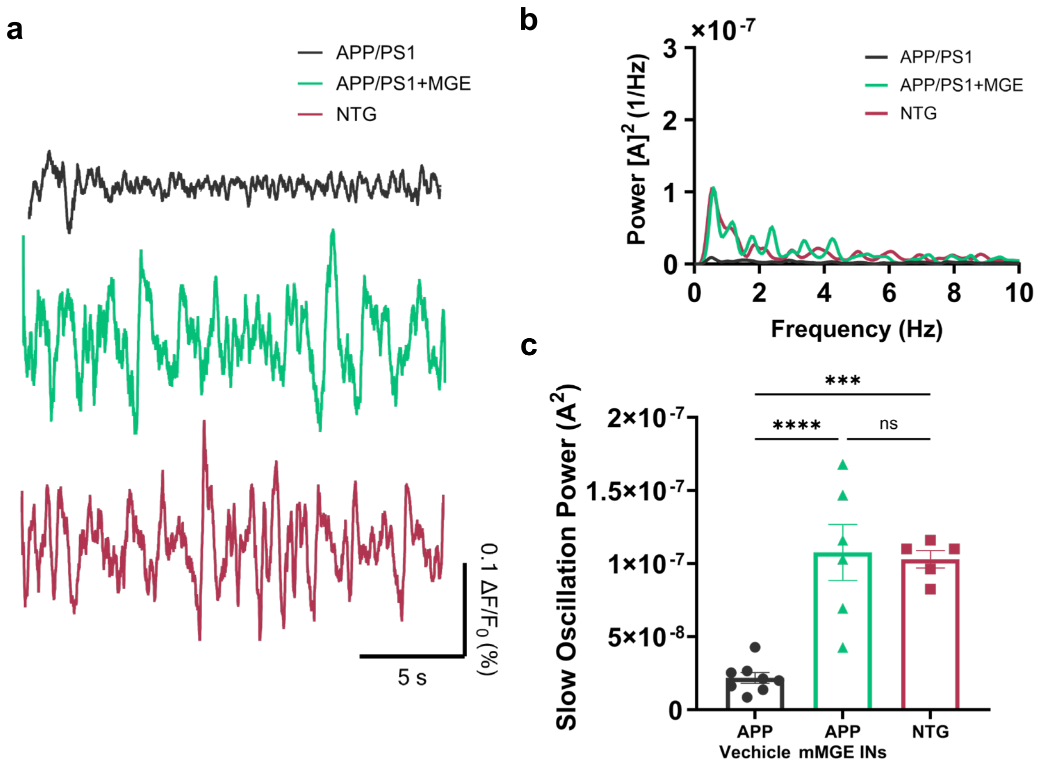

Supplementary Fig. 4 | MGE transplantation in APP/PS1 mice restores slow oscillation power to a level comparable to that of NTG mice. a Raw fluorescence traces from the APP/PS1 (gray), MGE transplanted APP/PS1 (green), and non-transgenic (NTG: red) cortex. **b** Power spectral density of APP/PS1 (gray), MGE transplanted APP/PS1 (green), and non-transgenic (NTG: red). $[A]^2$ = magnitude of Fourier amplitude squared. **c** Slow oscillation power. Each data point represents the average of 10–24 traces from each mouse. Data are presented as mean ± SEM, ns, not significant, $***p < 0.001$, $****p < 0.0001$. n = 5-7 mice/group.

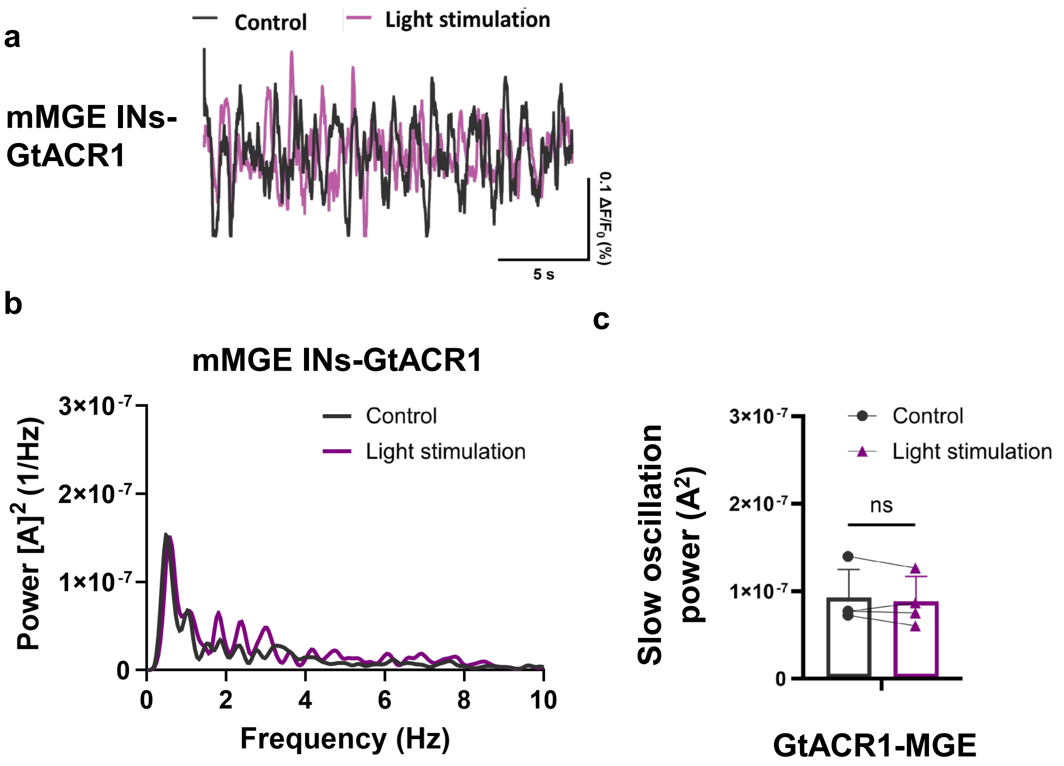

Supplementary Fig. 5 | Random light stimulation of transplanted MGE cells does not affect slow oscillation power. a Raw fluorescence traces from the host APP/PS1 cortex during random light stimulation (purple) and no stimulation (gray). **b** Power spectral density of MGE-transplanted APP/PS1 cortex with or without optogenetic stimulation $[A]^2$ = magnitude of Fourier amplitude squared. **c** Slow oscillation power with or without optogenetic light stimulation. Each data point represents the average of 10–15 traces from each mouse. Data are presented as mean ± SD, with ns indicating no significant difference. $n = 4$ mice/group.

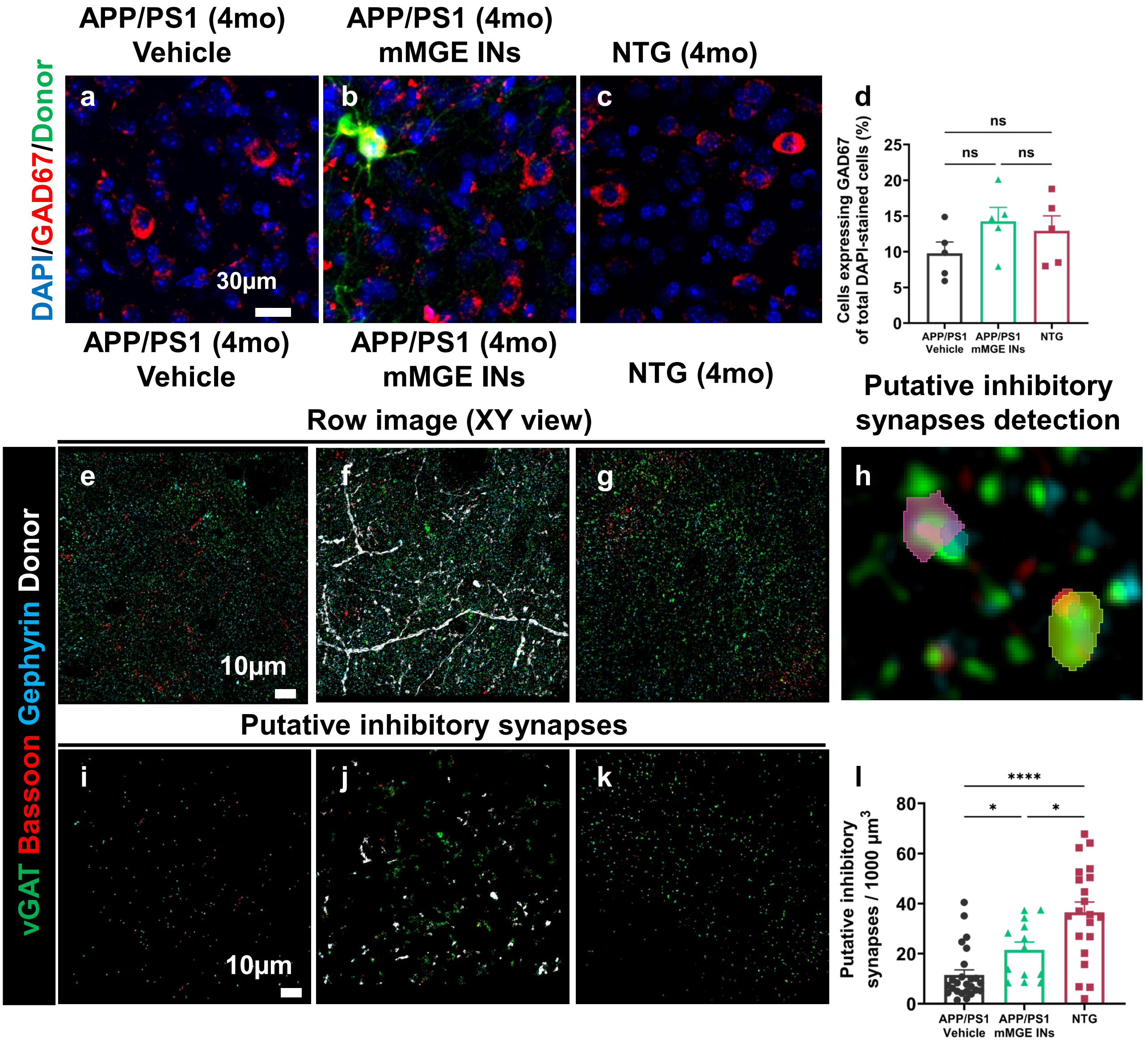

Supplementary Fig. 6 | MGE Transplantation partially restored inhibitory synaptic densities 2 months post-transplantation. a–c GAD67 immunostaining in the host anterior neocortex. **d** Quantification of GAD67 positive cells per DAPI stained cells. n = 5 mice/group. Each data point represents the average of more than 3 ROIs. Data are presented as mean ± SEM. The ordinary one-way ANOVA was applied. ns, not significant. **e–g** SIM images of synaptic marker labeling (VGAT, Bassoon, Gephyrin) in the host anterior neocortex. **h** Putative inhibitory synapse detection using the Blob Finder operation and filtering. Object filters have been applied as follows: bassoon objects must be inside vGAT objects or have >50% overlap with them; gephyrin objects must be attached to or overlapping bassoon objects. **i-k** Distribution of putative inhibitory synapses. **l** Quantification of synaptic density (per volume). n = 13-25 ROIs from 5 mice per group. Data are presented as mean ± SEM. Brown-Foreythe and Welch's ANOVA was applied. $*p < 0.05$, $****p < 0.0001$, Scale bars: 400 μm (**a**), 50 μm (**c, i**), 20 μm (**m**), and 100 μm (**o**).
